# Breakdown of Scaling and Friction Weakening in Intermittent Granular Flow

**DOI:** 10.1038/s41598-019-53178-2

**Published:** 2019-11-18

**Authors:** A. Baldassarri, M. A. Annunziata, A. Gnoli, G. Pontuale, A. Petri

**Affiliations:** 1 CNR - Istituto dei Sistemi Complessi, Dipartimento di Fisica, Università di Roma Sapienza, P.le A. Moro 2, I-00185 Roma, Italy; 2 Consiglio per la Ricerca in Agricoltura e l’Analisi dell’Economia Agraria (CREA) - Research Centre for Forestry and Woods, Via Santa Margherita 80, I-52100 Arezzo, Italy

**Keywords:** Physics, Statistical physics, thermodynamics and nonlinear dynamics, Phase transitions and critical phenomena

## Abstract

Many materials are produced, processed and stored as grains, while granularity of matter can be crucial in triggering potentially catastrophic geological events like landslides, avalanches and earthquakes. The response of grain assemblies to shear stress is therefore of utmost relevance to both human and natural environment. At low shear rate a granular system flows intermittently by distinct avalanches. In such state the avalanche velocity in time is expected to follow a symmetrical and universal average behavior, whose dependence on the slip size reduces to a scale factor. Analyzing data from long lasting experiments, we observe a breakdown of this scaling: While in short slips velocity shows indeed a self-similar and symmetric profile, it does not in long slips. The investigation of frictional response in these different regimes evidences that this breakdown can be traced back to the onset of a friction weakening, which is of dynamical origin and can amplify instabilities exactly in this critical state, the most frequent state for natural hazards.

## Introduction

The way a granular medium responses to an applied shear stress reveals many of the peculiarities of this poorly comprehended “state” of matter^1^. When a granular bed is sheared slowly enough by an elastic medium driven at constant velocity, nor the shear stress neither the shear rate can be directly controlled from outside. Rather, the system sets itself in a state at the edge between jamming and mobility^2–9^, exhibiting intermittent flow also called stick-slip. This is an instance, among many others, of phenomena displaying intermittent and erratic activity, in the form of bursts, or *avalanches*, characterized by wild fluctuations of physical quantities also called *crackling noise*^10^. Examples include earthquakes^11^, fractures^12^, structural phase transitions^13^, plastic deformation^14^, and other diverse phenomena sharing several common statistical features. In particular, these phenomena often display long range correlations and self-similar distributions, i.e. power laws, of physical quantities over a wide range of values. In equilibrium physical systems such properties are usually observed in the vicinity of a critical phase transition. Therefore also in non equilibrium systems they are usually ascribed to the vicinity of some critical point^10,15^, which in granular media is identified with the jamming transition^16^. Consistently, critical transitions bring about the existence of universality classes, where systems microscopically very different display similar and universal statistical properties in their critical, i.e. self similar, dynamics.

Many features of the crackling noise, i.e. intermittency, and broad time and energy scales, are produced in simple laboratory experiments, where small beads of glass are slowly sheared by an elastic medium^3^. Such experiments not only supply relevant information for a better comprehension of the irregular dynamics of granular matter, but can also help to discriminate between critical systems, yielding important elements for the general understanding of off-equilibrium dynamics.

In order to compare different systems exhibiting critical dynamics, several quantities can be analyzed. Recent literature witnesses a surge of interest for the average avalanche time profile, or shape^17^. Introduced in the context of Barkhausen noise in ferromagnetic materials^18^, the average avalanche shape can provide a much sharper tool to test theory against experiments than the simple comparison of critical exponents characterizing probability distributions^19^. As shown for simple stochastic processes, the geometrical and scaling properties of the average shape depend on the temporal correlations of the dynamics^20–22^. Average avalanche shapes have been investigated for a variety of materials, well beyond magnetic systems^17,23^, ranging from intermetallic compounds and crystals^24,25^ to glassy and amorphous systems^19,26–28^ and, very recently, in cortical bursts^29,30^, in transport processes in living cells^31^, as well in ants^32^ and in human^33^ activity. Burst shape has also been investigated in stellar processes^34^, Earth’s magnetospheric dynamics^35^, and earthquakes^36^.

We have acquired for the first time the *slip velocity* and the *friction force* in a sheared granular system, directly in the stick-slip phase, where it displays intermittent flow, and analyzed the corresponding average time profiles for different slip duration. In granular systems, the average time profile of velocity is expected to be symmetrical and invariant up to a rescaling^37^. On the contrary, in our study we observe the existence of a crossover from small slips, whose shapes satisfy the above properties, to large, non rescalable and non symmetrical slips. We identify this transition as a breakdown of the critical scaling, and show that the crossover corresponds to a characteristic speed marking a dynamical transition from weakening to thickening frictional behavior. These findings supply new essential elements to the formulation of novel and accurate dynamical models, with important impact on the understanding of related natural and technological issues. They also shed light on some apparently contradictory recent observations concerning avalanches of stress drop^37^ and energy drop rate^38^, conducted in continuous  flow. While Denisov *et al*.^37^ observed avalanches displaying symmetrical and self-similar average shapes, Barés *et al*.^38^ found these properties only in sufficiently small avalanches. The investigation reported here, conducted in the intermittent state, shows that symmetric and asymmetric slips can both exist, depending on their duration and as a consequence of the rheological properties of the system.

## Results

### Scaling analysis

In our experiments (see Methods and Supplementry Information) an assembly of glass spheres laying in an annular channel is sheared by a horizontally rotating top plate. The plate is driven by a motor through a soft torsion spring. Although the angular speed of the motor is kept constant, the interaction between the plate and the granular medium is crucial in determining the instantaneous plate velocity. The intermittent dynamics, in which the plate performs highly irregular and intermittent motion, is approached when both driving speed and spring constant are low enough.

**Figure 1:**
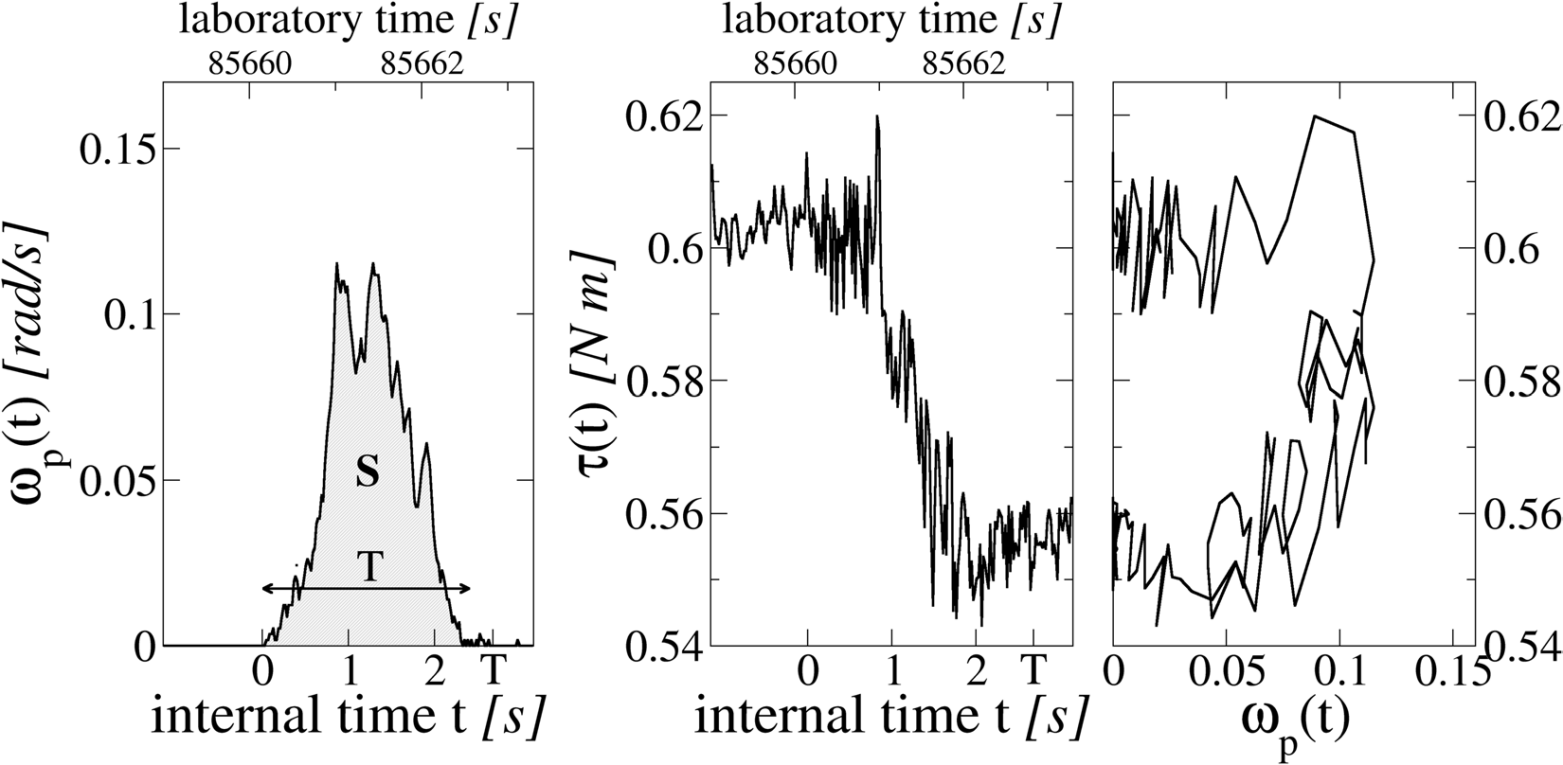
Sample of raw data for one of the six thousand slip events used in the present analysis. Left: instantaneous velocity of the slider versus time. The upper axis of the graph reports the total time elapsed from the beginning of the experiment, while the bottom axis indicates the internal avalanche time, starting from 0 when the slip begins, and ending at slip duration *T*. The area below the curve is the total slip size *S*. Center: Friction torque experienced by the slider in the same time window. Right: Behavior of friction torque vs instantaneous slider velocity for the same slip.

We have performed long lasting experimental runs in the intermittent, stick-slip regime, measuring the time behavior of the angular coordinate of the plate *θ*_*p*_(*t*) and the plate angular velocity *ω*_*p*_(*t*). We have then analyzed the stick-slip dynamics of the single slip event, illustrated in Fig. 1. The left panel reproduces the angular velocity during a slip. The motion can be described as a function of the *internal avalanche time t*, which starts at the beginning of the slip and ends when the system sticks. Each slip has its own duration *T* and size *S* (the grey area). The middle panel shows the corresponding frictional torque experienced by the plate in the same time interval. The right panel reports the instantaneous torque as function of the instantaneous plate velocity, showing how intricate and complex can be the relation relating these two quantities. Computing the average time profile of the velocity, or shape, requires to consider many slips of the same total duration *T* and to perform averages as function of the internal avalanche time *t*, as described below. In a similar fashion one can consider the average friction time shape, i.e. the time behavior of the torque exerted in average by the granular medium on the plate at the internal time *t* during a slip event of total duration *T*.

**Figure 2:**
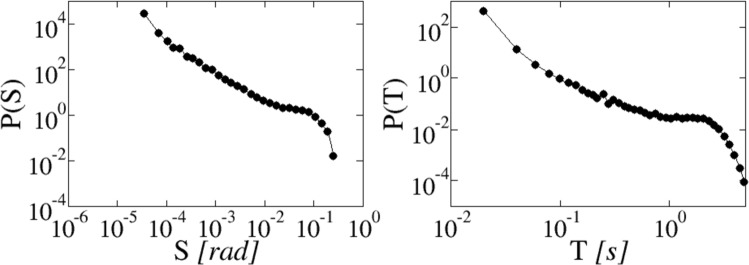
Probability distribution of slip extensions *S* (left) and durations *T* (right).

We have collected statistics from a large number of avalanches. The distribution of corresponding durations *T* and sizes $S={\int }_{0}^{T}\,{\omega }_{p}(t)dt$ are shown in Fig. 2. Both distributions exhibit a slow decay, close to a power law and terminating with a bulging cutoff at large values. Similar broad distributions are shared by other quantities^4^ like the plate velocity.

As recalled in the introduction, power law decays in distributions are generally considered the hallmark of critical phenomena. In this scenario, one expects to observe self-similar scaling relations in average quantities too. In particular, one can consider the average time profile of the velocity during an avalanche of duration, *T*:

$${\langle {\omega }_{p}(t)\rangle }_{T}=\frac{1}{{N}_{T}}\sum _{i}\,{\omega }_{p}^{(i)}(t),$$

where *ω*_*p*_^(*i*)^ is the plate velocity during the *i*_*th*_ observed avalanche of duration *T*, whose total number is *N*_*T*_, and *t* is the internal time within the slip: 0 < *t* < *T*. Although the average velocity profile 〈*ω*_*p*_(*t*)〉_*T*_ depends on both *t* and *T*, criticality^19,39,40^ implies that an invariant function Ω exists, such that it can be expressed as:

1 $${\langle {\omega }_{p}(t)\rangle }_{T}=g(T)\Omega (t/T),$$

where the function *g*(*T*) determines how the average event size 〈*S*〉_*T*_ scales with respect to the slip duration *T*. This scaling scenario is produced by several theoretical models of critical dynamics^19^. One paradigmatic model is the so called ABBM model^41^, proposed to describe the intermittent statistics of the electric noise induced during the hysteresis loop of ferromagnetic materials (Barkhausen noise), and simple enough to allow exact analytical results^22,23,41–43^. It predicts power law distributions for avalanche size and duration, as well as parabolic average avalanche shape in the scaling regime, and has inspired the extant models for intermittent granular flow^4,44^. We will discuss the connections between this model and what we observe in our study in the conclusive section.

### Average time profile of the slip velocity

**Figure 3:**
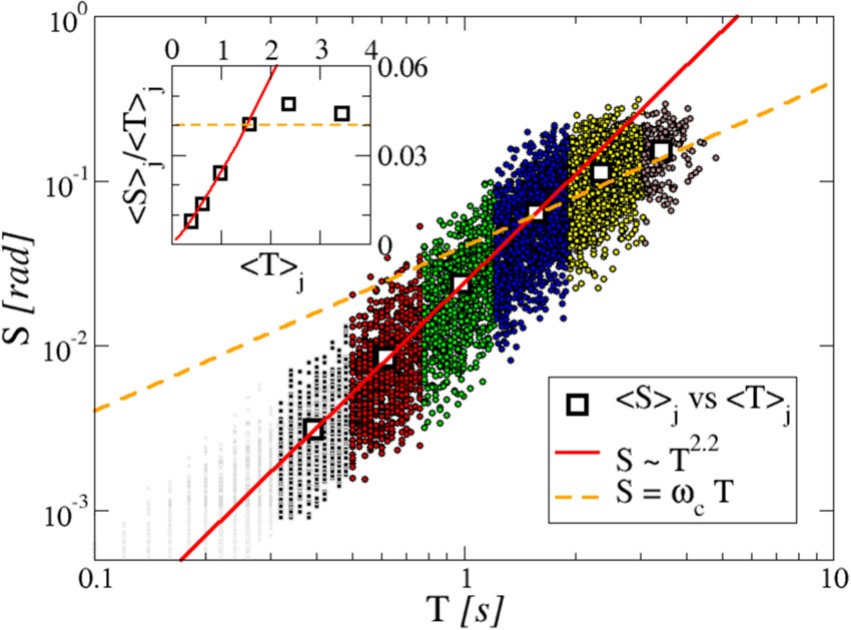
Scatter plot of size *S* vs duration *T*: each small point represents a single slip event. The events are divided into six classes of increasing duration, each one indicated with a different symbol and color (for the precise definition of the classes see SI). For each class *j* = 1, …, 6, the average duration 〈*T*〉_*j*_ and an average slip 〈*S*〉_*j*_ are computed, represented as white squares in the middle of each class. Inset: Average slip velocity 〈*S*〉_*j*_/〈*T*〉_*j*_ for each class as a function of average duration 〈*T*〉_*j*_ of the class. Lines (both in main plots and in inset), are guide to the eyes for: power law behavior (continuous line) *S* ≈ *T*^2.2^, and linear behavior (dashed line) *S* = *ω*_*c*_*T* (where *ω*_*c*_ = 0.04, see text and Fig. 5 for definition).

**Figure 4:**
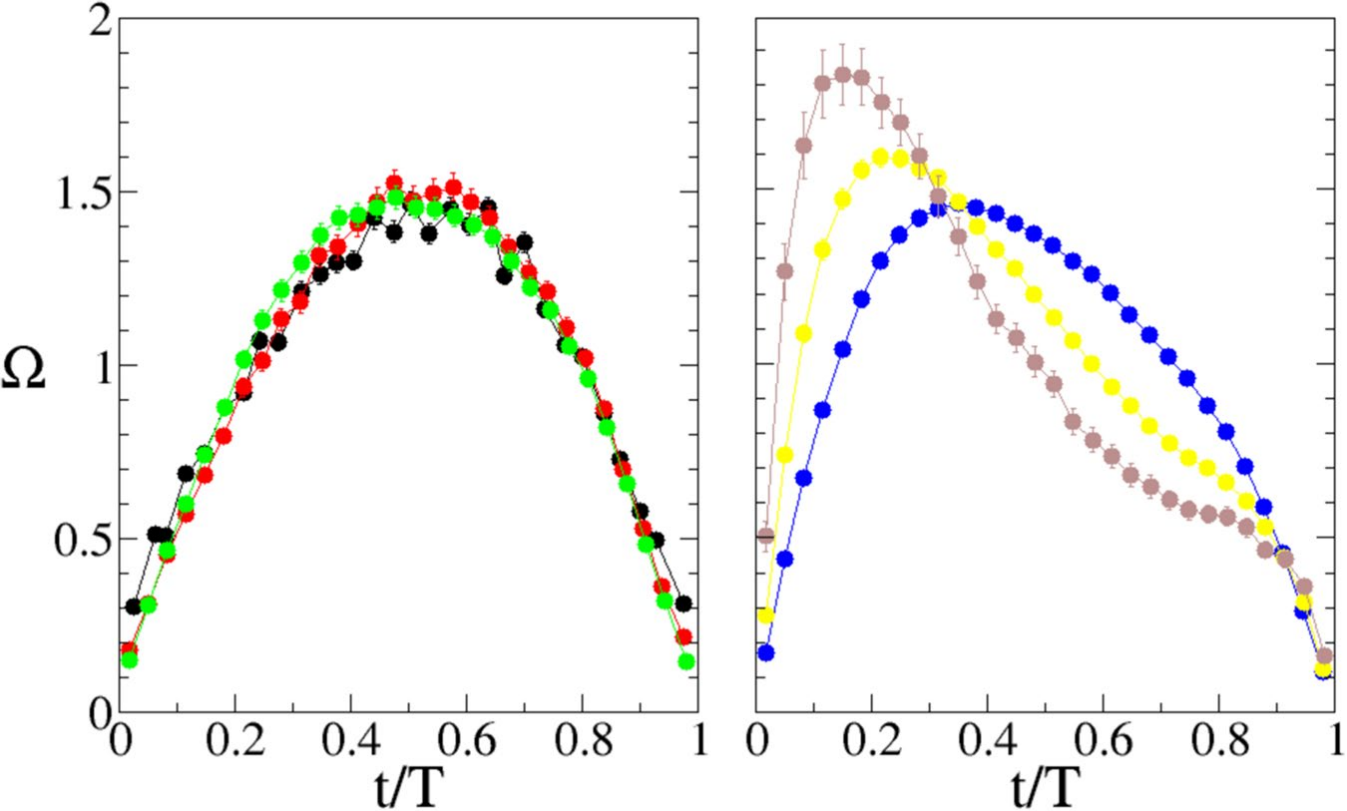
Time profiles of the normalized average velocity profile during a slip for “short” and “long” avalanches, as a function of the internal time *t*/*T* (Eqs 1 and 4). Average profiles are computed over avalanches belonging to a same class of duration, shown in the scatter plot of Fig. 3 with the same symbol coding (see SI for the precise definition). The left panel shows the average velocity profile for “short” avalanches (first three classes), and their very good scaling, while the right panel shows the profile for “long avalanches” (last three classes), where the scaling is broken.

To investigate the properties of the average time profile of the velocity, and to test the hypothesis of scaling invariance, we have divided all the avalanches observed in the experiments into classes according to their duration (see Mehods and Supplementry information). Figure 3 (main panel) shows the size of each slip as function of its duration, for all the slips considered in the statistics. Different colors and symbols correspond to the different classes of duration. For each class we have computed the rescaled average velocity time profile, or shape Ω of Eq. 1 (see Methods and Supplementry information). The resulting profiles are shown in Fig. 4 (light, gray points in Fig. 3, corresponding to very short slips at the limit of the system resolution, have not been considered). In the scaling scenario Ω is a function of *t*/*T* only, and it is seen that all classes exhibit comparable values of the rescaled maximum velocity, implying that longer avalanches are also faster. However, average shapes unveil that there are two kinds of avalanches. Some of them, say *short*, have the shape described by a unique function Ω(*t*/*T*), visible in Fig. 4 (left panel). That is, their rescaled velocity profile and duration are related by the well defined scaling law Eq. 1. On the contrary, the average velocity shapes of *long* avalanches (right panel) change with the duration and cannot be reduced to a universal form by a homogeneous rescaling of the variables. Moreover, they do not display the almost symmetric shape characterizing small avalanches.

As anticipated, Barés and coworkers^38^ have recently measured the average shape of stress drop rate avalanches in a bidimensional granular system driven at constant shear rate. Similarly to the present findings, they have observed that longer slips develop left asymmetries. They have hypothesized a possible role of the static friction between particles and supporting glass, and of nonlinear elasticity, given by the relatively soft nature of the grain material employed in their experiments. We can however exclude these factors in our experiments, where the interface grain-wall is small with respect to the bulk and the beads are made of a rather stiff material like glass. The leftwards asymmetry observed in experiments represents a genuine and very interesting phenomenon, which cannot be due to the simple inertia of the moving plate (which should produce opposite asymmetry^20,21^) and originates from non trivial dynamical effects.

### Breaking of scaling

More insight into the mechanisms leading to the scaling breakdown can be gained by further analyzing the scatter plot relating *S* and *T* in Fig. 3. The first information coming from this plot is that there exists a definite statistical scaling between slip size and duration. The white squared symbols in the main plot represent the average slip size and duration of each class (statistical errors are negligible on these averages). It is seen that for the four lower classes they follow an algebraic relation: ${\langle S\rangle }_{j}\simeq {\langle T\rangle }_{j}^{\alpha }$ (red continuous line). The value of the exponent turns out to be $\alpha \simeq 2.2$ (which is close to, but clearly different from the value of *α* = 2 expected from extant models, as for instance the ABBM model mentioned above^41^). The second information supplied by the scatter plot of Fig. 3 is that this behavior changes for long slips, where a linear dependence, 〈*S*〉 ∝ 〈*T*〉 looks more appropriate (yellow dashed line). Notice that the crossover between the two behaviors takes place around the fourth class, exactly where the scaling of the average pulse shape breaks down in Fig. 4. This fact is put into better evidence in the inset of Fig. 3, where we have plotted the average velocity of the whole slip 〈*S*〉_*j*_/〈*T*〉_*j*_, as function of 〈*T*〉_*j*_. Here a weakly superlinear relation for short slips is followed by a plateau at long slips. This allows to identify a characteristic velocity *ω*_*c*_, as the ratio between the average slip size and the average duration of the fourth class. It results *ω*_*c*_ = 〈*S*_*c*_〉/〈*T*_*c*_〉 ≈ 0.063/1.57 ≈ 0.04 rad/s. The slowdown of large avalanches suggests an increase of friction in long slips (*T* > *T*_*c*_), when the plate reaches velocities *ω*_*p*_ > *ω*_*c*_. In the next section it will be seen that indeed this increase appears as a dynamical effect.

### Stochastic friction

Friction in granular systems is usually measured under controlled shear strain or stress. The classical Mohr–Coulomb criterion predicts constant friction at low shear, and increasing values when the system enters the Bagnold’s regime^45,46^, a behavior well observed experimentally at constant shear (see e.g.^47^). However, it is doubtful whether this description applies to the stick-slip dynamics exemplified in Fig. 1. As a matter of fact, in the stick-slip regime, friction is a random quantity. Fluctuations in the frictional response of the granular medium result from the stress propagation along the evolving network of grain contacts, and are at the very origin of the motion stochasticity. Some statistical features of friction in this state have been investigated^3–5^, but despite this quantity plays a crucial role for the system dynamics, it seems that it has never been systematically measured during stick slip to date.

**Figure 5:**
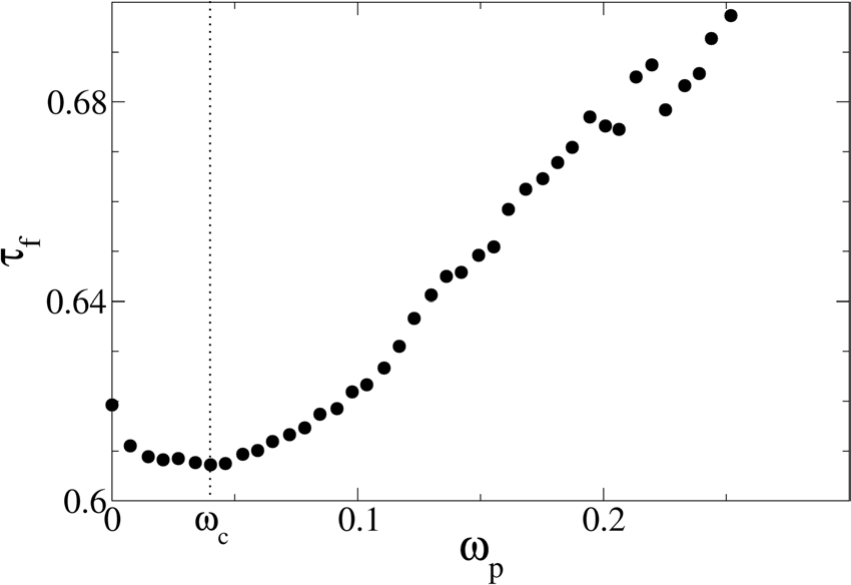
Experimental measure of the average friction torque at a given instantaneous plate velocity *ω*_*p*_ (defined in Eq. 5). The curve displays weakening behavior before reaching a minimum value at a characteristic velocity *ω*_*c*_ ≈ 0.04 rad/s, after which friction increases for increasing plate velocities.

A random friction force, as a stochastic quantity, can be described by statistical estimators like averages, moments, correlators, etc. One can consider for example the time average of the friction over the full dynamics, but this does not look really meaningful. Another possibility^4,48^ is to consider the average friction as function of the instantaneous plate velocity (see Methods). We plot such conditioned average friction, *τ*_*f*_, during the stick slip regime, in Fig. 5. An interesting weakening of friction at small velocities can be observed, followed by a thickening which recovers the Bagnold behavior at high velocities, a behavior also known as Stribeck curve. This velocity weakening arises as a pure dynamical effect since at constant shear the average friction is constant at low and intermediate speeds^47^.

Figure 5 allows to identify a velocity corresponding to the position of the minimum of the average friction *τ*_*f*_. It is always attained near the *ω* ≈ 0.04 rad/s, and our experiments clearly indicate that the position of this minimum does not depend on the drive velocity (see figures in the SI). This value is very close to the value *ω*_*c*_ marking the crossover in the scaling of *S* vs *T* in Fig. 3, and the breakdown of shape self-similarity in the average avalanche (Fig. 4), reinforcing the idea that both originate from the different friction experienced by the plate during longer, faster avalanches.

**Figure 6:**
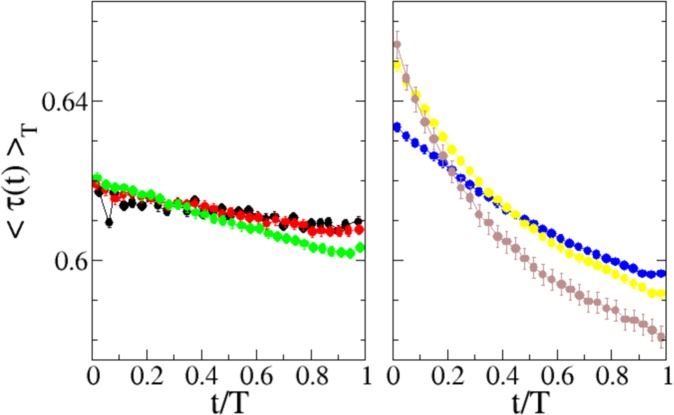
Experimental measure of the average friction torque along slips of different duration, as function of the internal time. The averages are performed grouping avalanches into classes according to their duration (see SI for the definition). Each curve refers to a different class shown with same colors and symbols used in Figs 3 and 4. Left panel shows the average friction for “short” avalanches (first three classes), while right panel shows the behavior for “long” avalanches (last three classes).

In order to better understand whether and how friction dynamical behavior can influence the average velocity shape, we have analyzed also the average shape of friction along the slip. In analogy to what has been done for computing the velocity shapes, one can define 〈*τ*(*t*)〉_*T*_ as the average frictional torque for slips of the same duration *T*. In practice, we have computed the average value of the friction torque during slips of similar duration *T*, according to the same classes of duration adopted for velocities (Fig. 3 and Supplementry information). The results are presented in Fig. 6 and show that to the break of the velocity shape scaling, it corresponds a change in the properties of the average friction shape. In scaling avalanches (left graph of Fig. 6) the average friction is largely independent from the slip duration and almost constant along the whole slip. On the contrary, the averages corresponding to longer slips (right graph of Fig. 6) display different shapes that, as for velocity (Fig. 4), strongly depend on *T* and cannot be collapsed. Moreover, in comparison with short slips, longer slips display higher friction at the beginning of the slip and lower at the end.

**Figure 7:**
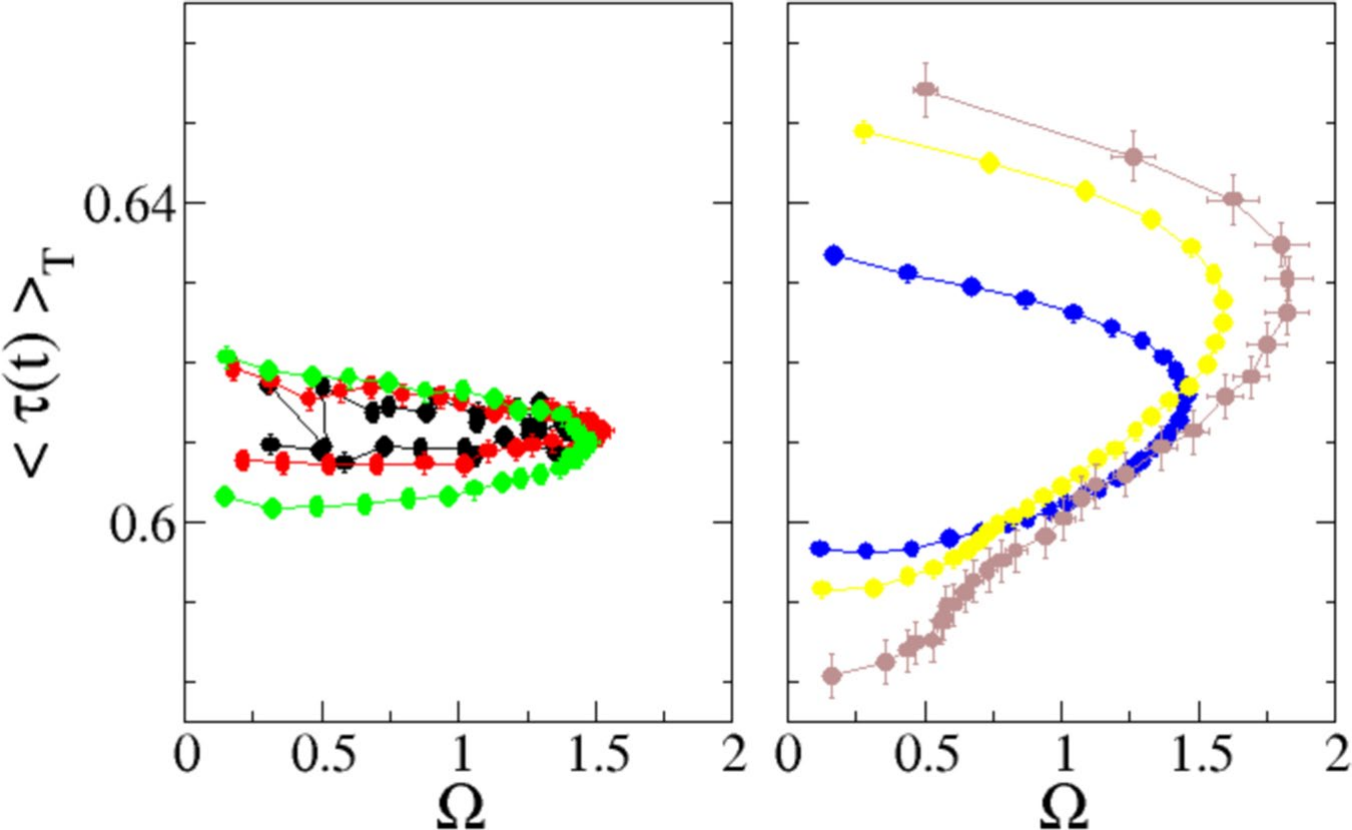
Average friction torque along slips of different duration as a function of the normalized average slip velocity. The plot is a parametric combination of Figs 4 and 6. The averages are performed over avalanches of similar duration, according to six classes of increasing duration (see SI for the definition). Each symbol refers to a different class withe the same coding used in Figs 3, 4 and 6. Left panel shows the behavior for “short” avalanches (first three classes), while right panel shows the behavior for “long” avalanches (last three classes).

Let us stress here the difference between the two averages considered above (Figs 5 and 6). The average 〈*τ*〉_*T*_ shown in Fig. 6, is performed over slips of similar duration, at the same internal avalanche time *t*. Instead, the (conditional) average *τ*_*f*_, defined in Eq. 5 and shown in Fig. 5, mixes events of any duration and depends on the instantaneous plate velocity *ω*_*p*_. The two quantities give different aspects of the same (stochastic) physical phenomenon. Nevertheless, the combination of the two analysis indicates that the quite complex friction weakening behavior of *τ*_*f*_ is mainly due to long slips, which show a non constant average friction 〈*τ*〉_*T*_ in time (Fig. 6, right panel) in contrast with small slips, where the average stays mainly constant. This point becomes still more evident by considering the curves resulting from combining the analysis of friction and velocity shapes, plotting the average friction as a function of the average velocity as shown in Fig. 7. It is seen that while in small slips (left panel), friction has a low velocity dependence (as could be expected from Fig. 6), in long ones it displays hysteresis, splitting into a two-valued function (right panel) with different dependency on velocity in the accelerating and decelerating phase, similarly to what observed in periodic stick-slip^49,50^ (where all slips have identical extension, duration, and velocity profile).

## Discussion

**Figure 8:**
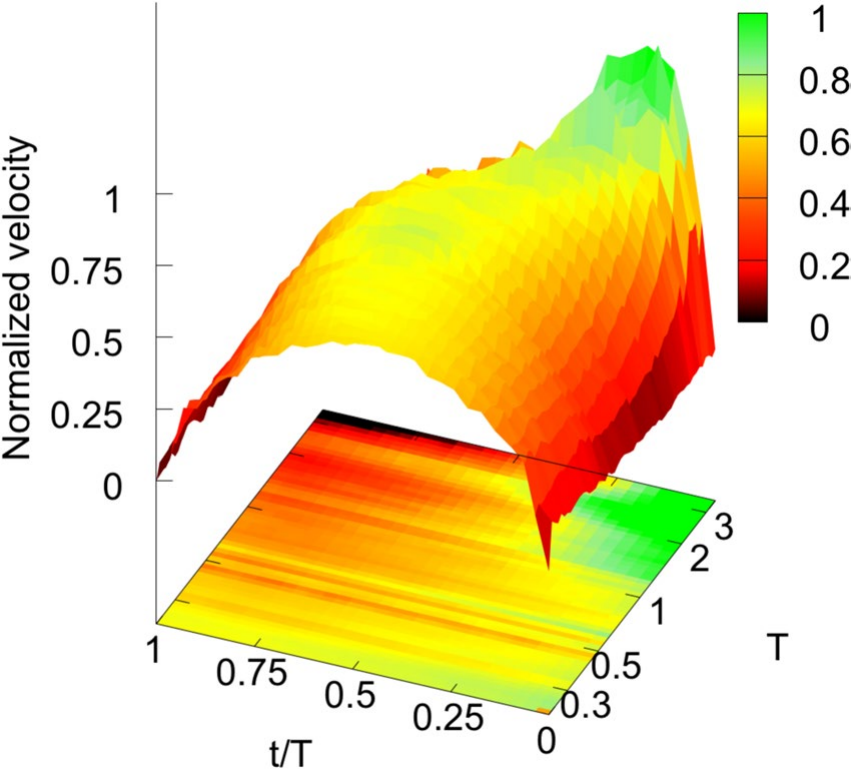
The figure shows the instantaneous velocity (upper surface) and the instantaneous friction (bottom plane) of each single slip observed in the experiment, as functions of the avalanche duration *T* and of its internal time *t*/*T*. The instantaneous velocity of each slip has been normalized by the average velocity during the slip, and then by a factor common to all slips such that the resulting velocities span between 0 and 1 (shown by the colors on the surface). Note how the “short” avalanches display a simmetric, scaling profile, while for “long” avalanches, the scaling is broken and the profiles get more and more asymmetric. The instantaneous friction, globally rescaled in order to get values spanning between 0 (minimum friction) to 1 (maximal friction), is represented with the color map in the botton plane. Note that the instantaneous friction for “short” avalanches keeps quite constant, while for “long” avalanches decreases markedly during the avalanches.

Our experiments show good scaling of the average velocity time profile in short avalanches, with an almost symmetric shape. In longer avalanches however, profiles take a clear leftward asymmetry and scaling, Eq. 1, is broken. A similar phenomenology has been observed also in a 2*d* system^51^, and in earthquakes^36,52^, where it is not yet clear the way different mechanisms can contribute to the shear weakening observed in coseismic fault shearing^53^. Our analyses show that the breakdown of scaling takes place along with a change in the friction behavior, pointing out a strict relation between the two phenomena. These results are summarized in Fig. 8, where all the slips of the present analysis are reported. For each slip the plot shows the instantaneous velocity (*z* surface) and the corresponding instantaneous friction (bottom map), as function of the internal time *t*/*T* and of the avalanche duration *T* (data have undergone a standard interpolation in order to get a smoother surface). For both quantities, velocity and friction, colors represent the relative magnitude, from black (minimum values) to green (maximum values). The *z* values of instantaneous velocity are normalized to the average velocity during the avalanche. It is seen that a relatively constant and moderate value of friction, colored from yellow to orange, corresponds to the symmetric shape of short avalanches. On the contrary, longer asymmetric avalanches are related to decreasing values (in time) of the friction. In particular, the lowest friction values (black) are typically recorded in the final part of long slips (*t*/*T* ≈ 1), while the initial part (*t*/*T* ≈ 0) displays the highest values (green).

The figure illustrates in a synthetic way the complexity of the frictional response of the granular bed, and highlights the necessity of considering suitable friction laws to model such phenomenology in effective way (spring-block models with imposed Coulomb friction generate symmetric slips^54,55^). On the other hand^19^, there is a strict relationship between avalanche shape symmetry and time reversibility, and this is in turn related to system non Markovianity, or memory. Friction laws working well for stationary flows^56^ cannot account for phenomena observed in non stationary situations, like for instance hysteresis. In the past, phenomenological friction laws accounting for the elapsed time and/or space along the slip have been derived from solid-on-solid experiments^57–59^. Although showing a limited validity for interstitial granular matter^60,61^, these *rate-and-state* laws and their simplified forms are often adopted for studying and modeling co-seismic fault shearing,^55,62–64^. Attempts of incorporating them to model stick slip experiments seems very rare^48^ and would be desirable.

An effective modeling approach to the stick-slip granular dynamics cannot as well exclude a stochastic description of friction, which generates slip unpredictability and variability, with the consequent change in the slip shapes. To our knowledge, the only few attempts in this direction^4,44,48^ are inspired to the aforementioned *ABBM model*^41^, which represents the mean field approximation for the motion of a driven elastic interface in a random environment^40,65^. From the dynamical point of view the model describes a spring–slider system in the overdamped approximation. (i.e. with negligible inertia) and subject to a friction which includes both a viscous force and a random pinning force. At small driving rate, the ABBM model produces intermittent, self-similar dynamics for the block motion. Similarly to our observations, avalanche statistics show a scaling regime for short slips, whose average velocity has parabolic shapes. However, an exponent *α* = 2 relates 〈*S*〉 to 〈*T*〉, which is different from what observed in our experiments. Moreover, for longer slips, ABBM predicts flatter but still symmetrical average velocity shapes, since no inertial effects are present. A variant of the ABBM model including inertia has shown to well reproduce the granular slip statistics^4^ in terms of extension, duration and velocities. Velocity distributions for an inertial ABBM model have also been studied by Le Doussal *et al*.^66^. Stimulated by the present results, investigations of the velocity shape generate by this model are in progress.

Finally, non trivial inertial effects should be considered. For instance, in some experiments^50^ an increased inertia of the slider moving on a granular bed was found, due to the grains dragged by the slider itself. In our previous experiments^4^ an effect of augmented inertia has been observed (in the scaling of the peak in the duration distribution). However, since during the irregular motion of the system the quantity of grains dragged by the disk could change, one should consider the inertia as a dynamical quantity, rather than a constant. The existence of such “effective” dynamical inertia of the system is another form of memory introduced by the underlying granular dynamics. An instance of such a mechanism, leading to a leftwards asymmetry in the avalanche shape, has been observed in some magnetic materials^67^ where it results from an effective negative mass of the domain walls. Grain inertia can influence the avalanche statistics even at the microscopic level. In sandpile models, largely studied in the context of Self Organized Criticality (SOC), the tendency of real sand grains to keep moving once they start facilitates the emergence of huge avalanches. This has been proven also experimentally, where small non inertial avalanches have been shown to have well distinct properties from large inertial ones^68,69^. Recent theoretical developments propose, in the presence of such facilitation effects, a scenario called Self-Organised Bistability (SOB)^70,71^, where again a breaking of scaling is associated to the appearance of large avalanches (“kings”).

In conclusion, our detailed scaling analyses show that symmetric and asymmetric average avalanche shapes are both present, but refer to different sizes, “short” vs “long”, and corresponding frictional regimes, explaining contradictory recent observations^37,38^. In addition, the study demonstrates that granular friction can exhibit weakening also in the vicinity of the jamming transition. Randomness and memory are general features that cannot be overlooked in the formulation of effective models of physical phenomena involving granular flow, as landslides and earthquakes, and of crackling noise in general.

## Methods

The experimental set up is described in detail in the SI and is similar to that employed in previous experiments^3–5,48,72^. It consists of an assembly of glass spheres laying in an annular channel and sheared by a horizontally rotating top plate driven by a motor. The instantaneous angular position of the plate and of the motor, respectively *θ*_*p*_ and *θ*_*d*_ are acquired by means of two optical encoders. The plate is coupled to the motor through a soft torsion spring of elastic constant *k*. The instantaneous frictional torque, *τ*, exerted by the granular medium can be derived from the equation of motion for the plate:

2 $$\tau =-\,k({\theta }_{d}-{\theta }_{p})-I{\ddot{\theta }}_{p},$$

where *I* is the inertia of the plate-axis system. The motor angular speed *ω*_*d*_ is kept constant, so that *θ*_*d*_(*t*) = *ω*_*d*_*t*.

To compute the average slip one can integrate Eq. (1) with respect to *t* obtaining:

3 $${\langle S\rangle }_{T}=Tg(T)$$

(where without loss of generality we have assumed ${\int }_{0}^{1}\,\Omega (x)dx=1$). The function Ω represents the average invariant pulse shape, which is expected not to depend on the slip duration and can be computed via the above equations as

4 $$\Omega (t/T)=T\frac{{\langle {\omega }_{p}(t)\rangle }_{T}}{{\langle S\rangle }_{T}}.$$

After classifying the slips according to their duration (as described in the subsection “Average shape of slip velocity” and in the SI), we have computed for each class, *j*, the average slip size 〈*S*〉_*j*_ and duration 〈*T*〉_*j*_, and the average velocity 〈*ω*_*p*_(*t*)〉_*j*_ measured as function of the internal time *t*. In order to obtain Ω(*t*/*T*), this average velocity has then been normalized to the ratio 〈*S*〉_*j*_/〈*T*〉_*j*_, according to Eq. 4.

The average friction as function of the instantaneous plate velocity *ω*_*p*_(*t*), discussed in the subsection “Stochastic friction” and shown in Fig. 5, has been computed as the average friction conditioned to the vaule of *ω*_*p*_(*t*):

5 $${\tau }_{f}(\omega )=\mathop{\mathrm{lim}}\limits_{T\to \infty }\frac{{\int }_{0}^{T}\,\tau (t)\delta (\omega -{\omega }_{p}(t))dt}{{\int }_{0}^{T}\,\delta (\omega (t)-{\omega }_{p})dt}.$$

## Supplementary Information

Supplementary information
