# Supplementary material for: Breakdown of Scaling and Friction Weakening in Intermittent Granular Flow
Source: Sci Rep. 2019 Nov 18;9:16962. doi: 10.1038/s41598-019-53178-2 (PMC6861274; doi:10.1038/s41598-019-53178-2)
Supplement: Supplementary file 1 — Supplementary information [file 41598_2019_53178_MOESM1_ESM.pdf]

Supplementary Information to

Breakdown of Scaling and Friction Weakening in the Intermittent Granular Flow

A. Baldassarri, M. A. Annunziata, A. Gnoli, G. Pontuale, A. Petri

Description of the experimental set-up

The experimental apparatus (Fig. S1) utilized for this research consists of a circular PPMI channel of outer and inner radii $R = 19.2$ cm and $r = 12.5$ cm respectively. The channel is 12 cm height and is almost filled with a bidisperse mixture 50%-50% of glass beads, with radii r_1=1.5 mm ± 10% and r_2=2 mm ± 10%.

A plate, fitting the channel, is set on the top. It can be rotated and has a few layers of grains glued to its lower face in order to better drag the underlying granular medium. The plate has mass $M = 1200$ g and moment of inertia $I = 0.026$ kg m^2 and is free to move vertically, implying that in our experiments the medium can change volume under a nominal pressure of $p = Mg/[\pi(R^2 - r^2)] \approx$176 Pa. It is connected to a torsion spring of

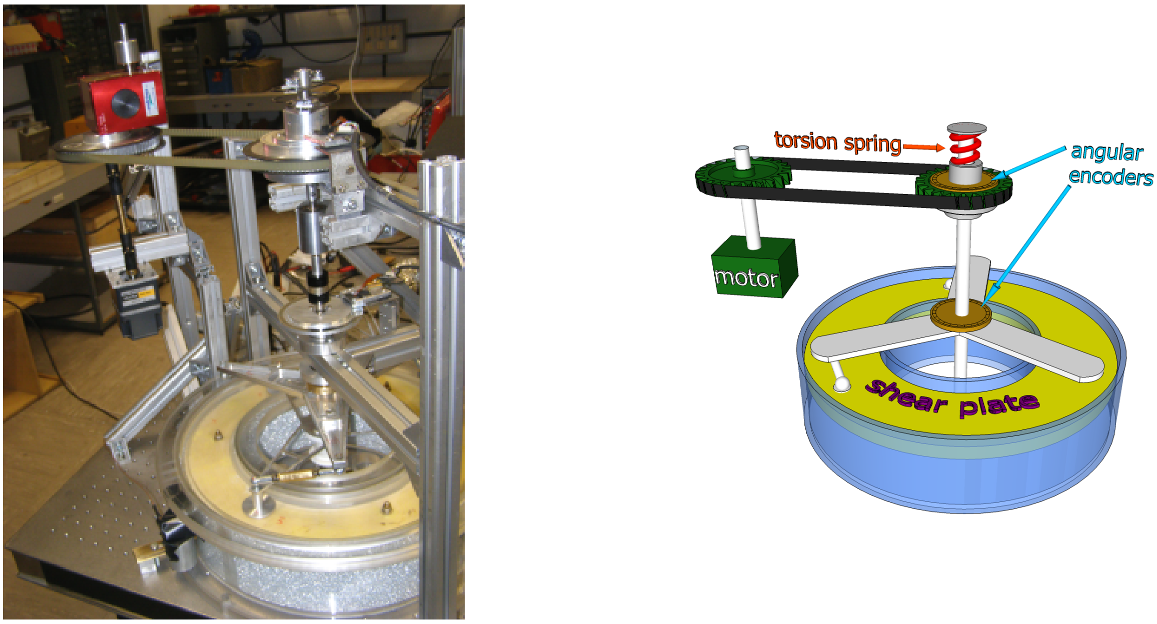

Figure S1: Photo (left) and schema (right) of the experimental set up.

elastic constant $\kappa = 0.36$ Nm/rad. The other end of the spring is connected to a motor

rotating at constant angular velocity ω_d. The angular positions of motor, θ_d, and plate, θ_p, are supplied by two optical encoders positioned on either side of the torsion spring, each one having a spatial resolution of $3 \cdot 10^{-5}$ rad and being sampled at 50 Hz. These measures also provide the instantaneous plate angular velocity ω_p and instantaneous acceleration $\dot{\omega_p}$, from which the instantaneous friction torque τ can be derived (Eq. 2 in the main text).

Experimental series

We have performed experiments with different driving speed ω_d. The adopted values are reported in Table S1. For each experiment the table reports also the duration, the number of data points of the acquired time series, and the number of avalanches employed in the statistics.

series	duration (minutes)	# of points	driving ω_d (rad/s)	# of slips used in analysis
(EA)	3900	5849962	0.0015	6014
(EB)	673	2020079	0.0022	1625
(EC)	1200	3600060	0.0044	5826
(ED)	4080	12240020	0.0055	2451
(EE)	360	1079977	0.011	3725
(EF)	240	720007	0.021	3973
(EG)	210	630014	0.033	4300

Table S1: Features of the analyzed series of experiments with different drives

The main text presents results from the series (EA). The results from the other data sets, with the different drives reported in Table S1, display similar behaviors and are shown in Figs. S2-S7, to be compared with the corresponding Figs. 2-7 in the main text. Analogous results were obtained adopting different sampling frequencies and threshold values.

Experimental analysis

In principle each single slip event, or *avalanche*, begins when ω_p starts to differ from zero and ends when ω_p goes back to zero. However, in practice it is necessary to choose a threshold value ω_{th} to cross, in order to get rid of the instrumental noise. This choice is to some extent arbitrary, however all the results have been observed to be independent from the chosen threshold, as long as it is enough small and different from zero. For our analysis we have set $\omega_{th} = 0.00175$ rad/s, and singled out the slips of the seven time series reported in Table S1.

The average shape of velocity during an avalanche of a fixed duration is defined as:

$$\langle \omega_p(t) \rangle_T = \frac{1}{N_T} \sum_i \omega_p^{(i)}(t),$$

duration	# of avalanches
$0.309 \leq T < 0.489$	929
$0.489 \leq T < 0.722$	866
$0.772 \leq T < 1.219$	987
$1.219 \leq T < 1.925$	1694
$1.925 \leq T < 3.04$	1380
$3.04 \leq T < 4.8$	158

Table S2: Classes of avalanche duration adopted for the analysis, and the resulting number of avalanches for the data set (EA) discussed in the main text.

where $\omega_p^{(i)}$ is the plate velocity during the i_{th} observed avalanche of duration T, whose total number is N_T, and t is the internal time within the slip: $0 < t < T$. The assumption is that a scale invariant function Ω exists, such that it can be expressed as:

$$< \omega_p(t) >_T = g(T)\Omega(t/T). \tag{S1}$$

The function $g(T)$ determines how the average event size $< S >$ scales with respect to the slip duration T. Integrating the above equation with respect to t one gets:

$$< S >_T = Tg(T) \tag{S2}$$

(where without loss of generality one assumes $\int_0^1 \Omega(x)dx = 1$) so that the function Ω can be computed via the above two equations as

$$\Omega(t/T) = T\,\frac{< \omega_p(t) >_T}{< S >_T}. \tag{S3}$$

To estimate the average shape Ω, the slips of each series have been grouped into classes on the base of their duration, according to the first column of Table S2. For each class j the instantaneous values of the plate velocity ω_p for each slip of the class have been averaged at a set of equispaced discrete times, $\{t_i\}$, such that $0 \leq t_i \leq \langle T\rangle_j$. Then, according to Eq. S3, $\Omega(t/T)$ has been computed by normalizing the obtained average velocities to the ratio $\langle S\rangle_j/\langle T\rangle_j$, where $\langle T\rangle_j$ and $\langle S\rangle_j$ are the average avalanche duration and size for the considered class j.

Avalanches at the extremes of the duration distribution have been dropped out when lacking resolution or statistics. Specifically, avalanches shorter than 0.31 s were too small to perform meaningful analysis (less than 15 points at 50Hz of sampling rate) and those longer than 4.8 s were usually too few. The number of avalanches employed for each class for the series (EA) is reported in the second column of Table S2.

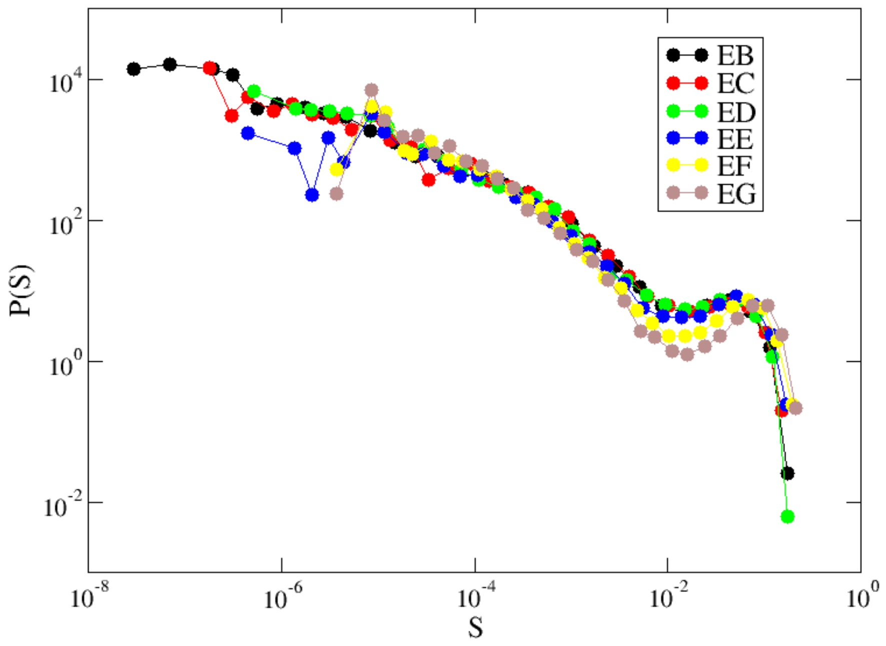

Figure S2: Avalanche size distributions for different drive velocities (see Fig. 2 in the main text).

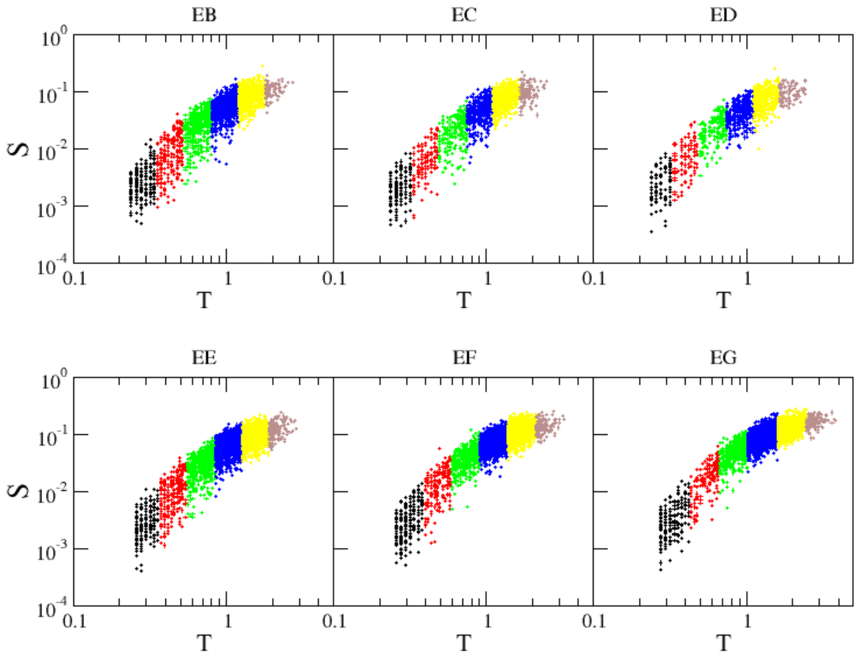

Figure S3: Avalanche sizes vs durations, and different classes from experiments with different drive velocities (see Fig. 3 in the main text).

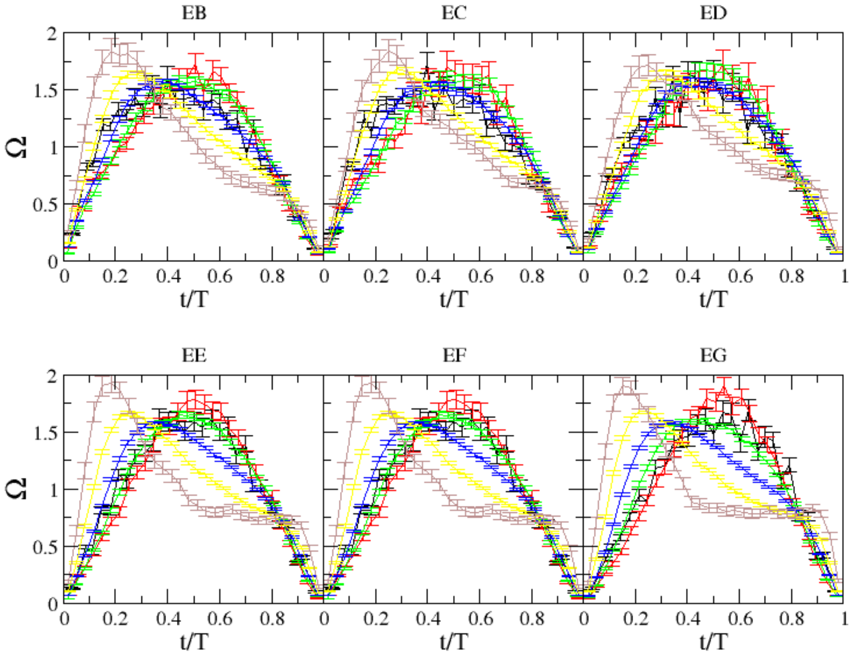

Figure S4: Average velocity shapes (see Fig. 4 in the main text) for the different drive velocities of Table S1.

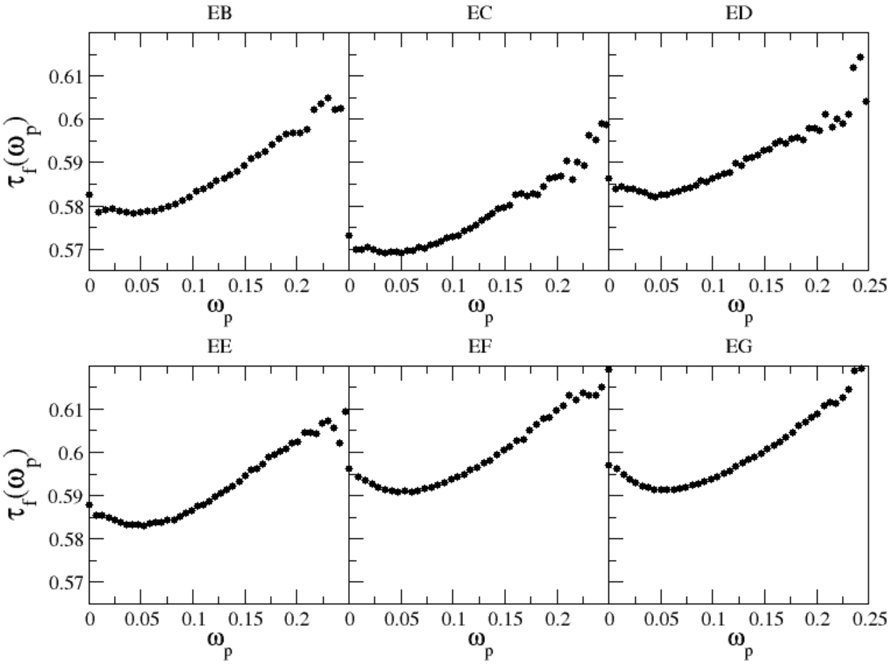

Figure S5: Conditional average friction vs instantaneous plate velocity (see Fig. 5 in the main text) for different drive velocities (Table S1).

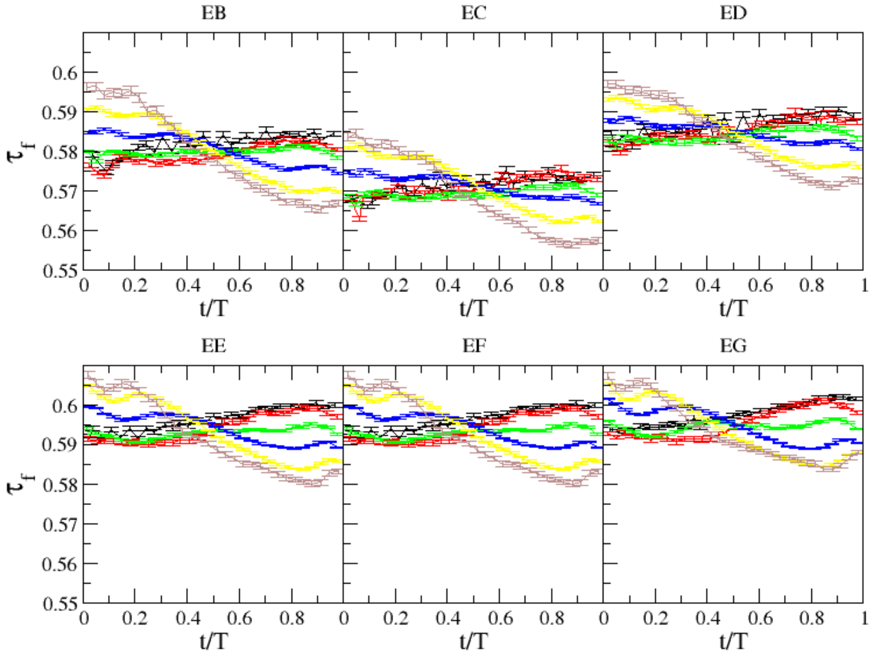

Figure S6: Average friction shapes (see Fig. 6 in the main text) for the different drive velocities of Table S1.

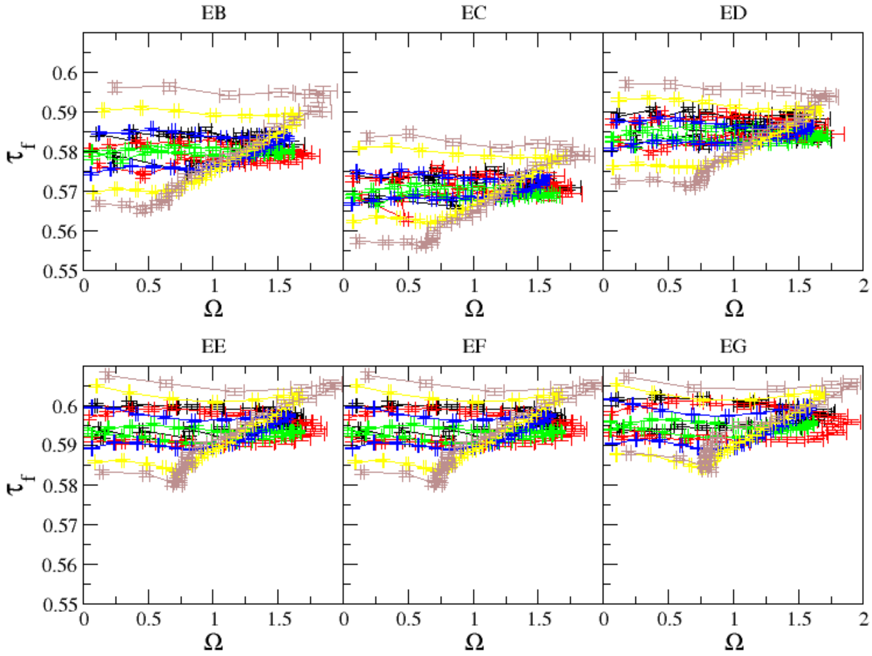

Figure S7: Average friction vs average rescaled velocity (see Fig. 7 in the main text) for different drive velocities (Table S1).
